# Expression of a novel splice variant of *FRMD7* in developing human fetal brains that is upregulated upon the differentiation of NT2 cells

**DOI:** 10.3892/etm.2014.1916

**Published:** 2014-08-19

**Authors:** YINGZHI LI, JIALI PU, BAORONG ZHANG

**Affiliations:** Department of Neurology, Second Affiliated Hospital, Zhejiang University School of Medicine, Hangzhou, Zhejiang 310009, P.R. China

**Keywords:** idiopathic congenital nystagmus, splice variant, *FRMD7*, neuronal development, retinoic acid

## Abstract

*FRMD7* mutations are associated with X-linked idiopathic congenital nystagmus (ICN); however, the underlying mechanisms whereby mutations of *FRMD7* lead to ICN remain unclear. In a previous study, the first *FRMD7* splice variant (*FRMD7-S*) was cloned and identified, and *FRMD7-S* was hypothesized to play a significant role in neuronal differentiation and development. The present study investigated a novel multiple exon-skipping mRNA splice variant of *FRMD7*, termed *FRMD7*_*SV2*, which was detected in NT2 cells using northern blotting. The mRNA expression levels of *FRMD7*_*SV2* in the developing human fetal brain were examined using reverse transcription polymerase chain reaction (PCR), while the expression levels in NT2 cells treated with retinoid acid (RA) or bone morphogenetic protein-2 were investigated using quantitative PCR. The results revealed that the expression of *FRMD7*_*SV2* was spatially and temporally restricted in human fetal brain development, and was upregulated upon RA-induced neuronal differentiation of the NT2 cells. These results indicated that as a novel splice variant of *FRMD7*, *FRMD7*_*SV2* may play a role in neuronal development.

## Introduction

Idiopathic congenital nystagmus (ICN) is an oculomotor disorder characterized by involuntary oscillation of the eyes, with an onset at birth or within the first six months of life and an estimated global prevalence of 24 in 10,000 births (1). ICN is genetically heterogeneous, and X-linked ICN is the most common mode of inheritance. Three genetic loci responsible for X-linked ICN have been mapped to chromosomes Xp11.3–p11.4 (2), Xp22 (3,4) and Xq26–Xq27. The FERM domain-containing protein 7 (*FRMD7*) at Xq26–Xq27 has been identified as responsible for X-linked ICN (5,6). Two novel missense mutations of the *FRMD7* gene in the Chinese population have been reported (7), and to date, >40 mutations have been identified (8,9). A previous study indicated that *FRMD7* may play an important role in neurite outgrowth, and downregulation of *FRMD7* results in a strong reduction of the neurite length (10). However, the biochemical role of *FRMD7* in neural development and the mechanisms whereby mutations of *FRMD7* lead to X-linked ICN remain unclear.

Alternative splicing produces multiple protein products with variable domain compositions from a single gene. The brain and testes show more prevalent alternative splicing compared with other tissues, indicating that these organs possess an unusually high number of splicing-associated genes (11–14). The *FRMD7* gene has been found to be subject to alternative splicing.

Previously, the first *FRMD7* splice variant, (*FRMD7-S*), containing a 45-bp truncation in the fourth exon (GenBank accession number, FJ717411), was cloned and identified. *FRMD7* and *FRMD7-S* were found to colocalize and coimmunoprecipitate. Furthermore, overexpression of *FRMD7* in NT2 cells resulted in altered neurite development and the upregulation of *FRMD7-S* (15). Based on these observations, a novel splice variant of human *FRMD7,* with missing exons 2, 3 and 4, was detected and a severely truncated protein was generated, termed *FRMD7*_splice variant 2 (*FRMD7*_*SV2*). The aim of the present study was to investigate whether *FRMD7_SV2* plays a role in neuronal development.

## Materials and methods

### Human embryonic brain tissue

Human fetal brain tissues at 14, 19 and 24 weeks post conception (wpc; gestational age was calculated from the first day of the last menstrual period; 14 wpc, n=2; 19 wpc, n=3; 24 wpc, n=2) were obtained from the Women’s Hospital affiliated to Zhejiang University School of Medicine (Hangzhou, China). All the samples were obtained and used in compliance with the Code of Ethics of the World Medical Association (Declaration of Helsinki) and the study was approved by the Second Affiliated Hospital of Zhejiang University School of Medicine (Hangzhou, China). Written informed consent was obtained from all the female participants of this study. The post-mortem interval for obtaining the samples was <6 h. Brain tissue samples (size, 1–3 mm^3^) were cut from each fetal brain, and the fresh tissues were stored in cryogenic vials (Corning; Sigma-Aldrich, St. Louis, MO, USA) containing liquid nitrogen. RNA extraction experiments were performed within three days.

### Cell culture, retinoic acid (RA)/bone morphogenetic protein-2 (BMP-2)-induced differentiation and immunofluorescence

Human NTERA-2/cl.Dl (NT2) cells were obtained from the Cell Culture Center of Peking Union Medical College (Peking, China) and cultured in Dulbecco’s modified Eagle’s medium/F12 (Gibco Life Technologies, Carlsbad, CA, USA), supplemented with 10% fetal bovine serum (FBS; HyClone™; Thermo Fisher Scientific, Waltham, MA, USA), 100 U/ml penicillin and 100 μg/ml streptomycin (Gibco Life Technologies). A stock solution (10 mM) of all-trans RA (Sigma-Aldrich Trading Co., Ltd., Shanghai, China) was prepared in dimethyl sulfoxide and stored at −75°C. Recombinant human BMP-2 was dissolved in normal saline (50 μg/ml) and stored at −20°C. Prior to the induction of differentiation, the NT2 cells were incubated (temperature, 37°C; high humidity; 5% CO_2_) in 25-cm^2^ cell culture flasks (Corning; Sigma-Aldrich) containing 4 ml culture medium.

In the differentiation experiments, the NT2 cells were divided into two groups: Group 1 was treated with RA (16,17), while group 2 was treated with BMP-2 (18,19). On day 0, the culture medium was replaced with Opti-MEM I (Invitrogen Life Technologies, Grand Island, NY, USA), containing 4% FBS and 10 μM RA (group 1) or 50 ng/ml BMP-2 (group 2). The cells were collected for RNA isolation after 12, 24 and 48 h incubation (early time points) or after 5, 8, 12 and 14 days (later time points). For prolonged cultures (>3 days), the culture medium was refreshed every three days in the continued presence of RA or BMP-2.

### RNA extraction, reverse transcription polymerase chain reaction (RT-PCR) and quantitative PCR (qPCR) analyses

Total RNA was extracted from the human embryonic brain tissue samples and NT2 cells using TRIzol reagent (Invitrogen Life Technologies), according to the manufacturer’s instructions. Total RNA (5 μg) and 1 μl oligo(dT)_18_ primer (0.5 μg/μl), with a total volume of ≤15 μl, were mixed in water. The test tube holding the mixture was heated to 70°C for 5 min and immediately immersed in ice-cold water for 5 min. Next, 1 μl M-MLV reverse transcriptase (200 U/μl; Promega Corporation, Madison, WI, USA), 5 μl M-MLV 5X reaction buffer, 2 μl dNTPmix (10 mM), 25 units recombinant RNasin^®^ ribonuclease inhibitor (Promega Biotech Co., Ltd, Beijing, China) and diethylpyrocarbonate water were added to the sample solution (total volume, 50 μl). The mixture was incubated for 1 h at 42°C, followed by 10 min at 70°C. For the PCR amplification, specific oligonucleotide primer pairs (10 pmol each) were incubated with 2 μl cDNA template in 25 μl PCR reaction mixtures, containing 2.5 μl 10X PCR buffer, 1.5 mM MgSO_4_, mixed deoxynucleotides (1 mM each) and 0.5 units KOD PLUS polymerase (Toyobo Corporation, Osaka, Japan). For amplification of the full-length *FRMD7* gene and its splice variant, PCR was performed for 40 cycles at 95°C for 2 min, 95°C for 20 sec, 56°C for 20 sec and 72°C for 150 sec, followed by a final elongation step at 72°C for 5 min. The primer sequences were as follows: P1-forward, 5′-ATGCTACATTTAAAAGTGCAGTTT-3′, and P1-reverse, 5′-TTAAGCTAAAAAGTAATTACATGGT-3′.

Relative gene expression levels were measured with qPCR on a Light Cycler™ Real-time PCR thermocycler (Roche Diagnostics Corporation, Basel, Switzerland), using SYBR^®^ Premix Ex *Taq*™ (Perfect Real Time; Takara Biotechnology Co., Ltd., Dalian, China). For the amplification of *FRMD7*_*FL*, the specific primers used were as follows: P2-forward, 5′-CAAAGCAGGTAAAAAATCCTAAGG-3′ [melting temperature (Tm), 62°C], and P2-reverse, 5′-ATGTGAGATACCATCAACGCTGT-3′ (Tm, 60°C). For the amplification of *FRMD7_SV2,* the following primers were used: P3-forward, 5′-CCAGAA GATTTTTGTGGTTGATGTAT-3′ (Tm, 70°C) and P3-reverse, 5′-GAGTTTGTGCCAGATGCTTCCTAT-3′ (Tm, 70°C). Each reaction amplified a single product, and all PCR amplifications were conducted in triplicate. The mean fold change in the expression of the target gene at each time point was calculated using the 2^−ΔΔCt^ method (20), with GAPDH as the endogenous control. The PCR products were run on 2% agarose gels to confirm the amplification size and identify the single PCR product.

### FRMD7 and FRMD7_SV2 detection in NT2 cells using northern blotting

Total RNA was extracted from NT2 cells that had been induced by RA, according to the manufacturer’s instructions (Ambion Life Technologies, Carlsbad, CA, USA). The total RNA was separated by electrophoresis on a 1% agarose gel in 1X TBE buffer [90 mM Tris-boric acid and 2 mM EDTA (pH 8.0)] and transferred to a nylon membrane (Amersham Biosciences, Amersham, UK). Following crosslinking under ultraviolet light, the membrane was prehybridized in DIG Easy Hyb Granules buffer (Roche Diagnostics Operations, Inc., Indianapolis, IN, USA) for 30 min. Subsequently, the membrane was hybridized with a DIG-labeled probe (*FRMD7* probe, 5′-TAC TGAGATGGGTAATGTTTCCTTTCAAATGGCAAGCTC TTCAG-3′) at 42°C overnight. A DIG-labeled actin probe (5′-CAAACATGATCTGGGTCATCTTCTC-3′) was used as the control, while RNA Molecular Weight Marker I labeled with DIG was used as the marker. Immunological detection was performed using the DIG High Prime DNA Labeling and Detection Starter Kit II (Roche Diagnostics Corporation), according to the manufacturer’s instructions.

### Statistical analysis

All the experiments were conducted in triplicate and the data are represented as the mean ± standard error of mean. Statistically significant differences between groups were identified using the Kolmogorov-Smirnov test and Student’s t-test, as implemented using SPSS 13.0 software (SPSS, Inc., Chicago, IL, USA). P<0.05 was considered to indicate a statistically significant difference.

## Results

### Identification of a novel alternative splicing variant of the human FRMD7 gene

**Figure 1:**
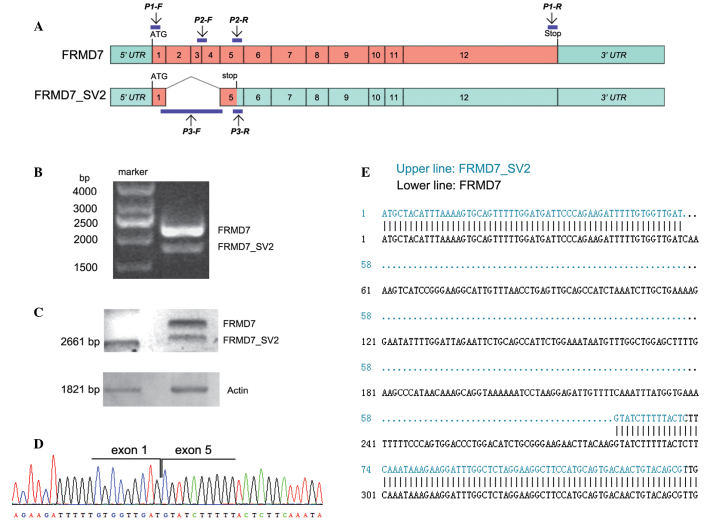
Cloning and identification of a splice variant of the *FRMD7* gene (*FRMD7_SV2*) and detection in NT2 cells with northern blotting. (A) Gene structure of *FRMD7_FL* and *FRMD7_SV2* and primer set location. (B) *FRMD7_FL* and *FRMD7_SV2* cDNA molecules were isolated from NT2 cell cDNA (synthesized from the total RNA of NT2 cells induced by RA for seven days). (C) *FRMD7_FL* (upper lane) and *FRMD7_SV2* (lower lane) were detectable in NT2 cells (total RNA of NT2 cells induced by RA for seven days) at the same time. Actin was used as the control. (D and E) Sequence comparison between *FRMD7_FL* and *FRMD7_SV2*. *FRMD7_FL,* full-length *FRMD7*.

To obtain the full-length human *FRMD7* (*FRMD7*_*FL*) cDNA, RT-PCR experiments were performed on the RNA extracted from the NT2 cells using a pair of specific primers. DNA fragments were isolated by electrophoresis and two distinct fragments were observed on the gel (Fig. 1B). Sequence analyses were performed and a multiple exon-skipping mRNA splice variant of *FRMD7* was identified, which was missing exons 2, 3 and 4, and was assumed to generate a severely truncated variant. The splice variant was termed *FRMD7*_*SV2* (Fig. 1C–E). This multiple exon-skipping event eliminated 227 nucleotides of the *FRMD7*_*FL* gene and resulted in a frameshift mutation that altered 19 amino acids prior to the premature termination at codon 39 (TGA), predicted to lead to the synthesis of a severely truncated protein based on the sequence (Fig. 1A).

### Expression of FRMD7_SV2 is spatially and temporally restricted in human brain development

A previous study revealed a restricted expression of *FRMD7* in the human embryonic brain and development of the neural retina (10). In addition, high expression levels of *FRMD7*_*FL* and *FRMD7-S* were found in 16-wpc human fetal cerebellum samples (15). In the present study, the expression levels of the three variants at the three stages (14, 19 and 24 wpc) in the development of the human fetal brain were detected by RT-PCR, using isoform-specific primers.

**Figure 2:**
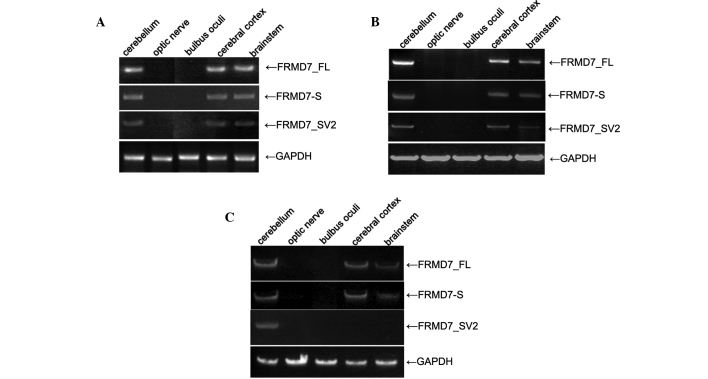
PCR analysis of *FRMD7_FL*, *FRMD7-S* and *FRMD7_SV2* in the developing human fetal brain, with the products separated by electrophoresis on 2% agarose gels. (A) At 14 wpc, the three variants were detected in the cerebral cortex, cerebellum and brainstem, but not in the optic nerve or bulbus oculi. (B) At 19 wpc, *FRMD7_FL* and *FRMD7-S* were detected in the cerebral cortex, cerebellum and brainstem, while the expression of *FRMD7_SV2* decreased slightly in the cerebral cortex and brainstem. (C) At 24 wpc, the expression of *FRMD7_FL* and *FRMD7-S* decreased in the brainstem, and *FRMD7_SV2* was only detected in the cerebellum. PCR, polymerase chain reaction; *FRMD7_FL,* full-length *FRMD7*; *FRMD7_S, FRMD7* splice; *FRMD7_SV2, FRMD7* splice variant 2; wpc, weeks post conception.

The results revealed that the mRNA expression levels of *FRMD7*_*FL* and *FRMD7-S* were high in the cerebral cortex, cerebellum and brainstem for all three stages; however, expression was not detected in the optic nerve or bulbus oculi. Similarly, *FRMD7*_*SV2* was expressed in the cerebral cortex, cerebellum and brainstem at 14 and 19 wpc; however, the expression level was slightly decreased in the cerebral cortex and brainstem at 19 wpc. At 24 wpc, *FRMD7*_*SV2* was only detected in the cerebellum (Fig. 2).

### mRNA expression levels of FRMD7_SV2 increase during RA-induced, but not BMP-2-induced differentiation of NT2 cells

To investigate whether *FRMD7*_*SV2* is involved in differentiation, the mRNA expression level of *FRMD7_SV2* in NT2 cells was examined during RA-induced or BMP-2-induced differentiation.

**Figure 3:**
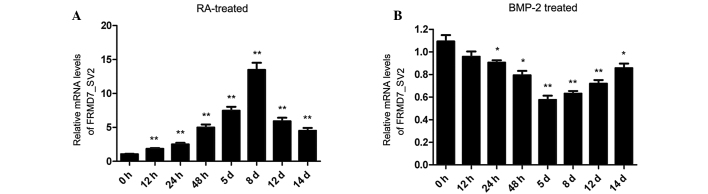
Expression levels of *FRMD7_SV2* in NT2 cells undergoing differentiation. qPCR analysis was performed to determine the relative mRNA expression levels of *FRMD7_SV2*. The experiments were conducted in triplicate. The day 0 sample (prior to the addition of RA) was used as the control, while cells treated with dimethyl sulfoxide/normal saline only were used as the negative control. Values are represented as the mean ± standard error of mean; ^*^P<0.05 and ^**^P<0.01, vs. 0 h group. (A) Relative expression levels of *FRMD7_SV2* increased significantly over time, with the highest expression level showing a 13.5-fold increase at day 8, followed by an evident decline. (B) mRNA expression levels of *FRMD7_SV2* in BMP-2-treated NT2 cells showed a small, but significant decreasing trend between 24 h and 14 days of BMP-2 treatment. qPCR, quantitative polymerase chain reaction; *FRMD7_SV2, FRMD7* splice variant 2; RA, retinoic acid; BMP-2, bone morphogenetic protein-2.

A time-course experiment was performed and the results revealed that the mRNA expression levels of *FRMD7*_*SV2* increased markedly during RA-induced neuronal differentiation of NT2 cells. Within 12 h of treatment with RA, the relative expression levels of *FRMD7*_*SV2* exhibited a 1.8-fold increase. The expression levels of *FRMD7*_*SV2* gradually increased over time, with the highest expression level increase (13.5-fold) detected within eight days of treatment with RA, in accordance with the developmental time course of neurite outgrowth. However, the expression levels of *FRMD7*_*SV2* showed an evident decline following eight days of treatment with RA (Fig. 3A). By contrast, *FRMD7_SV2* expression levels exhibited a small but significant decreasing trend between 24 h and five days of treatment with BMP-2, which stopped at day 5 and subsequently began to increase (Fig. 3B).

## Discussion

Alternative splicing is an established mechanism for gene diversification and increasing the complexity of mammalian transcriptomes. International genome and transcript sequencing projects have shown that the frequency of alternative splicing is associated with organism complexity, with up to 94% of human multi-exon genes alternatively spliced (21). The significance of alternative splicing is evident in highly specialized nerve cells, and highlighted in a number of neurological disorders (22).

In the present study, a novel *FRMD7* splice variant was identified through RT-PCR and qPCR analyses, and was termed *FRMD7*_*SV2*. This splice variant of human *FRMD7* was missing exons 2, 3 and 4, and presumably encoded a severely truncated protein. *FRMD7*_*SV2* eliminated 227 nucleotides of the full-length *FRMD7* gene and resulted in a frameshift mutation, altering 19 amino acids prior to premature termination at codon 39 (TGA). These changes were hypothesized to lead to the synthesis of a severely truncated protein based on the sequence.

Alternative splicing may change the structure of transcripts and their encoded proteins, determining the binding properties, intracellular localization, enzymatic activity, protein stability and post-translational modifications of a large number of proteins (23). Typically, alternative splicing shows tissue- and/or development-specific distribution, resulting in different expression levels in different cell lines or developmental stages (24). A previous study indicated that the COOH-terminus of *FRMD7* plays a key role in the subcellular localization of *FRMD7* in Neuro-2A and HEK 293T cells (25). In the present study, the splicing event occurred within the NH_2_-terminal FERM domain, and the resulting loss of the COOH-terminus of *FRMD7* may alter the function of the full-length *FRMD7* protein.

*FRMD7* is a member of the FERM family that causes X-linked ICN. Initial studies using *in situ* hybridization and immunohistochemistry revealed that the expression of *FRMD7* is spatially and temporally regulated in the human brain during embryonic and fetal development (10). In the present study, RT-PCR analysis was used to detect the expression of *FRMD7*_*SV2* mRNA in the developing human fetal brain (14, 19 and 24 wpc), and the results revealed that the expression level of *FRMD7*_*SV2* was also spatially and temporally restricted. At 14 and 19 wpc, *FRMD7*_*SV2* was detected in the cerebral cortex, cerebellum and brainstem; however, the expression level was slightly decreased in the cerebral cortex and brainstem at 19 wpc. At 24 wpc, *FRMD7*_*SV2* was only detected in the cerebellum. The cerebellum is involved in the coordination and precision of voluntary motor movement, balance and equilibrium and muscle fine-tuning functions. The vestibulocerebellum primarily regulates balance and spatial orientation. Any damage in this area causes disturbances in balance and gait (26), as well as nystagmus (27,28). These results indicate that the expression of the *FRMD7*_*SV2* gene exhibits spatially and temporally restricted distribution in the human fetal brain, indicating that the majority of *FRMD7*_*SV2* functions are associated with cerebellum development.

In a previous study, Betts-Henderson *et al* demonstrated that the mRNA and protein expression levels of mouse *FRMD7* were elevated during RA-induced differentiation of Neuro-2A neuroblastoma cells (10). Due to the lack of specific primer set data, it is unclear whether only the full-length *FRMD7* was detected or a combination of two or more transcripts. The NT2 cell line is a characterized human embryonic carcinoma cell line, and NT2 cells can be induced to differentiate into postmitotic central nervous system neurons when treated with RA (16,17). In addition, the cell line differentiates into non-neural epithelial lineages when treated with BMP-2 (18,19). Therefore, NT2 cells offer a valuable model for observing different trends in mRNA expression levels during RA-induced and BMP-2-induced differentiation processes. qPCR analysis revealed that the expression levels of the *FRMD7*_*SV2* gene gradually increased in RA-induced differentiating NT2 cells. A 13.5-fold increase in the expression level was detected within eight days of treatment with RA, in accordance with the developmental time course of neurite outgrowth. Therefore, *FRMD7*_*SV2* is hypothesized to be involved in the early stages of RA-induced neural differentiation in NT2 cells.

Previous observations provide evidence that *FRMD7* plays a critical role in neuronal morphogenesis, synapse function and neurite growth. Therefore, *FRMD7*_*SV2* is hypothesized to play a role in the function of *FRMD7*; however, further studies are required to confirm this hypothesis.

In summary, *FRMD7*_*SV2* was identified as a splice variant of the *FRMD7* gene. *FRMD7*_*SV2* may play a role in neuronal development and provide further evidence on the function of *FRMD7*.

## References

[1] Sarvananthan N, Surendran M, Roberts EO (2009). The prevalence of nystagmus: the Leicestershire nystagmus survey. Invest Ophthalmol Vis Sci.

[2] Cabot A, Rozet JM, Gerber S (1999). A gene for X-linked idiopathic congenital nystagmus (NYS1) maps to chromosome Xp11.4–p11.3. Am J Hum Genet.

[3] Schiaffino MV, Bassi MT, Galli L (1995). Analysis of the OA1 gene reveals mutations in only one-third of patients with X-linked ocular albinism. Hum Mol Genet.

[4] Bassi MT, Schiaffino MV, Renieri A (1995). Cloning of the gene for ocular albinism type 1 from the distal short arm of the X chromosome. Nat Genet.

[5] Tarpey P, Thomas S, Sarvananthan N (2006). Mutations in FRMD7, a newly identified member of the FERM family, cause X-linked idiopathic congenital nystagmus. Nat Genet.

[6] Schorderet DF, Tiab L, Gaillard MC (2007). Novel mutations in FRMD7 in X-linked congenital nystagmus. Mutation in brief #963. Online. Hum Mutat.

[7] Zhang B, Liu Z, Zhao G (2007). Novel mutations of the FRMD7 gene in X-linked congenital motor nystagmus. Mol Vis.

[8] Shiels A, Bennett TM, Prince JB, Tychsen L (2007). X-linked idiopathic infantile nystagmus associated with a missense mutation in FRMD7. Mol Vis.

[9] Thomas S, Proudlock FA, Sarvananthan N (2008). Phenotypical characteristics of idiopathic infantile nystagmus with and without mutations in FRMD7. Brain.

[10] Betts-Henderson J, Bartesaghi S, Crosier M (2010). The nystagmus-associated FRMD7 gene regulates neuronal outgrowth and development. Hum Mol Genet.

[11] Wang ET, Sandberg R, Luo S (2008). Alternative isoform regulation in human tissue transcriptomes. Nature.

[12] de la Grange P, Gratadou L, Delord M, Dutertre M, Auboeuf D (2010). Splicing factor and exon profiling across human tissues. Nucleic Acids Res.

[13] Li Q, Lee JA, Black DL (2007). Neuronal regulation of alternative pre-mRNA splicing. Nat Rev Neurosci.

[14] Venables JP (2002). Alternative splicing in the testes. Curr Opin Genet Dev.

[15] Li Y, Pu J, Liu Z (2011). Identification of a novel FRMD7 splice variant and functional analysis of two FRMD7 transcripts during human NT2 cell differentiation. Mol Vis.

[16] Andrews PW (1984). Retinoic acid induces neuronal differentiation of a cloned human embryonal carcinoma cell line in vitro. Dev Biol.

[17] Pleasure SJ, Page C, Lee VM (1992). Pure, postmitotic, polarized human neurons derived from NTera 2 cells provide a system for expressing exogenous proteins in terminally differentiated neurons. J Neurosci.

[18] Chadalavada RS, Houldsworth J, Olshen AB (2005). Transcriptional program of bone morphogenetic protein-2-induced epithelial and smooth muscle differentiation of pluripotent human embryonal carcinoma cells. Funct Integr Genomics.

[19] Caricasole A, Ward-van Oostwaard D, Zeinstra L (2000). Bone morphogenetic proteins (BMPs) induce epithelial differentiation of NT2D1 human embryonal carcinoma cells. Int J Dev Biol.

[20] Livak KJ, Schmittgen TD (2001). Analysis of relative gene expression data using real-time quantitative PCR and the 2(−Delta Delta C(T)) method. Methods.

[21] Johnson JM, Castle J, Garrett-Engele P (2003). Genome-wide survey of human alternative pre-mRNA splicing with exon junction microarrays. Science.

[22] Dredge BK, Polydorides AD, Darnell RB (2001). The splice of life: alternative splicing and neurological disease. Nat Rev Neurosci.

[23] Stamm S, Ben-Ari S, Rafalska I (2005). Function of alternative splicing. Gene.

[24] Yeo G, Holste D, Kreiman G, Burge CB (2004). Variation in alternative splicing across human tissues. Genome Biol.

[25] Pu J, Li Y, Liu Z (2011). Expression and localization of FRMD7 in human fetal brain, and a role for F-actin. Mol Vis.

[26] Martin JH, Cooper SE, Hacking A, Ghez C (2000). Differential effects of deep cerebellar nuclei inactivation on reaching and adaptive control. J Neurophysiol.

[27] Dieterich M, Brandt T (2008). Functional brain imaging of peripheral and central vestibular disorders. Brain.

[28] Harris CM, Walker J, Shawkat F (1993). Eye movements in a familial vestibulocerebellar disorder. Neuropediatrics.

